# Non-Invasive Assessment of Vascular Damage Through Pulse Wave Velocity and Superb Microvascular Imaging in Pre-Dialysis Patients

**DOI:** 10.3390/biomedicines13030621

**Published:** 2025-03-04

**Authors:** Julia Martín-Vírgala, Beatriz Martín-Carro, Sara Fernández-Villabrille, Belinda Fernández-Mariño, Elena Astudillo-Cortés, Minerva Rodríguez-García, Carmen Díaz-Corte, José Luis Fernández-Martín, Carlos Gómez-Alonso, Adriana S. Dusso, Cristina Alonso-Montes, Manuel Naves-Díaz, Sara Panizo, Natalia Carrillo-López

**Affiliations:** 1 Metabolismo Óseo, Vascular y Enfermedades Inflamatorias Crónicas, Instituto de Investigación Sanitaria del Principado de Asturias (ISPA), 33011 Oviedo, Spain; 2 Unidad Funcional de Metabolismo Óseo, Unidad de Gestión Clínica de Medicina Interna, Hospital Universitario Central de Asturias, 33011 Oviedo, Spain; 3 RICORS 2040-RENAL, 33011 Oviedo, Spain; 4 Área de Radiodiagnóstico, Hospital Universitario Central de Asturias, 33011 Oviedo, Spain; 5 Unidad de Gestión Clínica de Nefrología, Hospital Universitario Central de Asturias, 33011 Oviedo, Spain; 6 Departamento de Medicina, Universidad de Oviedo, 33011 Oviedo, Spain; 7 Division of Endocrinology, Metabolism and Lipid Research, Washington University School of Medicine, St. Louis, MO 63110, USA

**Keywords:** adventitial neovascularization, aortic stiffness, non-invasive, pulse wave velocity, superb microvascular imaging

## Abstract

**Background/Objectives**: Cardiovascular disease is the main cause of morbidity and mortality in Chronic Kidney Disease (CKD), so it is of great importance to find simple and non-invasive tools to detect vascular damage in pre-dialysis CKD patients. This study aimed to assess the applicability of non-invasive techniques to evaluate vascular damage in stages CKD-2 to CKD-5 and its progression after an 18-month follow-up using (A) carotid–femoral pulse wave velocity (PWV) to assess aortic stiffness and (B) Superb Microvascular Imaging (SMI) ultrasound to assess adventitial neovascularization compared with other traditional techniques to evaluate vascular damage, such as carotid intima–media thickness and Kauppila index. **Methods**: The study involved 43 CKD patients in stages CKD-2 to CKD-5 and a group of 38 sex- and age-matched controls, studied at baseline and at an 18-month follow-up. Age, sex, body mass index, arterial pressure, pharmacological treatments, and blood and urinary parameters were collected. Aortic stiffness was determined by carotid–femoral PWV and abdominal aortic calcification was assessed in lateral lumbar X-rays and quantified by the Kauppila index. Carotid intima–media thickness (cIMT), the number of carotid plaques, and adventitial neovascularization were evaluated by SMI. **Results:** Vascular impairment was mostly detected in CKD-4 and CKD-5 stages, with increased aortic stiffness measured by PWV and increased carotid plaques and adventitial neovascularization measured by SMI ultrasound. Furthermore, CKD-5 patients showed greater abdominal aortic calcification. Interestingly, CKD patients displayed a negative correlation between serum soluble Klotho (sKlotho) and cIMT. Finally, CKD patients showed no progression of vascular impairment after the 18-month follow-up, with the exception of carotid plaques. **Conclusions**: Performing non-invasive PWV and SMI ultrasound might be useful to evaluate vascular damage in CKD before entering dialysis, possibly helping to prevent cardiovascular events, although future studies should clarify the use of these techniques in clinical practice.

## 1. Introduction

Cardiovascular disease is the leading cause of premature death in Chronic Kidney Disease (CKD) patients undergoing dialysis [1]. Vascular impairment plays an important role in the CKD–Mineral Bone Disorder (CKD-MBD). Mineral metabolism is altered, so the levels of several regulators—phosphorus, calcium, parathyroid hormone (PTH), fibroblast growth factor 23 (FGF23) and soluble klotho (sKlotho), among others—lead to vascular calcification [2]. In turn, vascular calcification is the main cause of arterial stiffness in CKD, leading to increased morbidity and mortality, especially during dialysis. In CKD, calcification of the media is the most frequent form of vascular calcification, which includes osteogenic differentiation of vascular smooth muscle cells prompting calcium phosphate deposition [2]. Vascular calcification is often diagnosed once already established. The two most common detection sites are the abdominal aorta through X-rays, the severity of which can be estimated by the Kauppila index (KI) [3], and coronary arteries by computed axial tomography [4].

Additionally, alterations in lipid metabolism and systemic inflammation trigger vascular damage in CKD, often leading to atherosclerosis [5]. Increased carotid intima–media thickness (cIMT) or carotid plaques are usually evaluated to assess the risk of atherosclerosis, mainly by ultrasound probes—which do not always allow the study of plaque structure—or invasive and expensive techniques, such as magnetic resonance, contrast enhanced ultrasound (CEUS), or angiography [6]. In CKD-MBD, mineral disorders often lead to plaque calcification, which alters its composition and stability, as well as being probably linked to vascular repair failure [7]. Unfortunately, there is still some controversy on the topic of plaque calcification’s role in plaque instability [8].

Most tools to assess vascular damage are invasive, expensive, or detect vascular damage when it is already established in the form of calcification or plaques. Despite being simple and reproducible to assess, increased cIMT and carotid plaques indicate already established vascular damage, which is challenging to reverse. As for the Kauppila index, it shows aortic calcification in a precise way, but it requires radiation. Hence, the need for new non-invasive and simple techniques to apply in CKD patients before dialysis is of great importance, as dialysis implies great cardiovascular risk. One example is carotid–femoral pulse wave velocity (PWV), the gold-standard tool to evaluate aortic stiffness. It is mostly used as a complementary test after assessing arterial blood pressure although it is not applied in daily clinical practice.

Recently, Superb Microvascular Imaging (SMI) was developed to precisely study small and slow blood flux without the need of a contrast agent, thanks to an algorithm that removes motion artifacts [9]. SMI has allowed the detection of adventitial neovascularization in the carotid and femoral arteries, which is considered a possible early marker of vascular damage [10].

PWV is sometimes used in the cardiology field to complement the measurement of arterial pressure [11], but to date, it is not applied in CKD patients, despite their high cardiovascular risk. As for SMI ultrasound, it has been used as a novel technique to help in the diagnosis of cancer disease [12], inflammation [13], plaque instability [14], among others, but never to detect adventitial neovascularization in pre-dialysis CKD. Therefore, this study aimed to assess vascular damage in patients in stages CKD-2 to CKD-5 and its progression after an 18-month follow-up. The applicability of two non-invasive techniques which have been barely used in CKD patients was evaluated: (A) carotid–femoral PWV to assess aortic stiffness and (B) SMI ultrasound to assess adventitial neovascularization compared with other traditional techniques to evaluate vascular damage, such as carotid intima–media thickness and KI.

## 2. Materials and Methods

### 2.1. Ethics Statement

The study was approved by the Comité de Ética de la Investigación con medicamentos del Principado de Asturias, protocol number 140/19 in compliance with the Declaration of Helsinki. All participants included in the study provided written informed consent.

### 2.2. Study Participants

The study involved 43 CKD patients (25 men and 18 women) from the Nephrology Unit (Hospital Universitario Central de Asturias, Spain). Sample size calculations were performed a priori based on previous data on PWV values between patients and controls [15], with an alfa = 0.050 and a power = 0.90. CKD patients were classified according to their estimated glomerular filtration rate (eGFR) in mL/min/1.73 m^2^, following the 2017 KDIGO guidelines [16], into four groups: CKD-2/3a, 45–89; CKD-3b, 30–44; CKD-4, 15–29; CKD-5, <15. Sex- and age-matched individuals from the general population were recruited as the control group (18 men and 20 women). The cohort was studied at baseline and after an 18-month follow-up. The average age at baseline was 66.7 ± 8.6 years in CKD patients and 66.5 ± 4.6 years in the controls.

The exclusion criteria were (a) diabetes mellitus, (b) abdominal aneurism or intermittent claudication, (c) previous carotid surgery, (d) concomitant immune-mediated disease or cancer diagnosis, (e) ongoing immunosuppressive treatment, (f) recent or current infection, or (g) pregnancy.

In the 18-month follow-up, 8 patients were lost due to cancer diagnosis, dialysis treatment, or drop out.

Age, sex, body mass index (BMI), arterial pressure, pharmacological treatments, and blood and urinary parameters were collected (Table 1). Creatinine, total proteins, calcium, and phosphorus were determined using cobas 702 equipment (Roche Diagnostics), PTH and calcidiol using an electrochemiluminescence immunoassay (ECLIA, Roche Diagnostics), intact FGF23 and calcitriol with a chemiluminescence immunoassay (CLIA, DiaSorin), and serum sKlotho by a specific ELISA assay (Immuno-Biological Laboratories, IBL).

### 2.3. Vascular Outcomes

Arterial stiffness was determined in the left side by carotid–femoral pulse wave velocity (PWV), using Complior Analyze (ALAM Medical). The procedure is characterized by its relative simplicity to perform and is based on the time interval required for the arterial pulse wave to propagate from the carotid to the femoral artery. Higher PWV values indicate greater arterial stiffness; a value above 10 m/s is considered to reflect cardiovascular risk [17]. The results are the average of three optimal measurements.

SMI ultrasound allows for the detection of microvascularization in a very precise way, as it is able to differentiate blood flow signals from overlapping tissue motion artifacts, removing background “noise” thanks to an algorithm. This way, low speed blood signals can be accurately detected [9]. As SMI is an ultrasound tool, any experienced radiologist would be able to perform it without much specific training. B-mode ultrasound using the probe Superb Microvascular Imaging (SMI) Ultrasound (Toshiba Aplio 500, Toshiba American Medical Systems USA, Inc, Tustin, CA, USA) was performed using a Toshiba-Aplio XG machine (Toshiba American Medical Systems USA, Inc, Tustin, CA, USA) to analyze: left carotid intima–media thickness (cIMT), number of carotid plaques (either cIMT < 1.5 mm or a focal thickening going over into the arterial lumen by at least 50% of the surrounding cIMT value), and adventitial neovascularization in the carotid and femoral arteries. Seven SMI images were taken for each participant and were quantified by two operators blinded to the study, using Image J software. Two SMI images from subjects with and without neovascularization are shown as examples. Adventitial neovascularization was identified as small-caliber blood flow, which originated at the adventitia and grew toward the inside of the vessel (Supplemental Figure S1).

Vascular calcification in the anterior and posterior walls of the aorta was evaluated using the semi-quantitative Kauppila index (KI) on a lateral radiograph at the level of lumbar vertebrae L1 to L4, using the following score: 0, no calcification; 1, 1/3 of the vertebral body length was calcified; 2, 2/3 of the vertebral body length was calcified; and 3, the whole length of the vertebral body was calcified [3].

All vascular measurements were performed by operators blinded to the study participants.

### 2.4. Statistical Analysis

The results are shown as median [interquartile range] or mean ± standard deviation according to data distribution. Statistical comparisons between groups were performed using Kruskal–Wallis test (non-parametric analysis) or ANOVA (parametric analysis) and Dunn or Tukey tests (to calculate specific differences between every group) as post hoc analysis. A multivariate analysis of vascular damage, as a dependent variable, with age, the rest of the vascular measurements, or with different bone and mineral metabolism parameters were carried out after adjusting by age, gender, BMI, and eGFR, when necessary. A *p* value lower than 0.05 was considered statistically significant. All calculations used the statistical analysis package R (4.1.1 version).

## 3. Results

### 3.1. Anthropometric, Clinical, and Biochemical Features

Table 1 shows, at baseline, the anthropometric, clinical, and biochemical parameters and the pharmacological treatments in CKD-2/3a to CKD-5 patients and the control group. In CKD patients, no statistical differences were found between the baseline study and the 18-month follow-up in any anthropometric, clinical, or biochemical parameters (Supplemental Table S1), except for increased serum levels of creatinine (*p* = 0.002), FGF23 (*p* = 0.01), and sKlotho (*p* = 0.001).

No differences were found in age, sex, or BMI between the control group and CKD patients, except for a different proportion of males and females among CKD stages (Table 1).

CKD patients displayed higher systolic (control: 132 [121–143.5] mmHg and CKD: 140 [132.2–158.5] mmHg; *p* = 0.009) and diastolic blood pressure (control: 74.6 ± 9.7 mmHg and CKD = 79.0. ± 13.4 mmHg; *p* = 0.004) (Table 1).

As expected, from CKD-2/3a to CKD-5, serum creatinine levels increased, along with reductions in eGFR and urinary creatinine and significantly higher proteinuria/urine creatinine ratio (Table 1). No changes were observed in serum calcium, while serum phosphorus increased exclusively in CKD-5. Calcitriol levels significantly decreased, starting in CKD-3b. In CKD progression, even though there was a non-significant decrease in serum calcidiol, the normal values in stage 2/3a became insufficient in stages 3b, 4, and 5 in spite of vitamin D supplementation. Serum PTH and FGF23 significantly increased in CKD patients, starting at CKD-3b, while serum sKlotho diminished progressively from stage CKD-2/3a (Table 1).

Pharmacological treatments showed a trend to increase with CKD progression, except for stage CKD-5, in which statins, antihypertensives, and vitamin D supplementation levels were similar to those in CKD-2/3a patients (Table 1).

### 3.2. Vascular Damage in CKD and Its Association with Serum CKD-MBD Markers

The progression of aortic stiffness was measured by PWV, aortic calcification was estimated by KI, and adventitial neovascularization was assessed by SMI ultrasound.

#### 3.2.1. Pulse Wave Velocity: Aortic Stiffness

**Figure 1:**
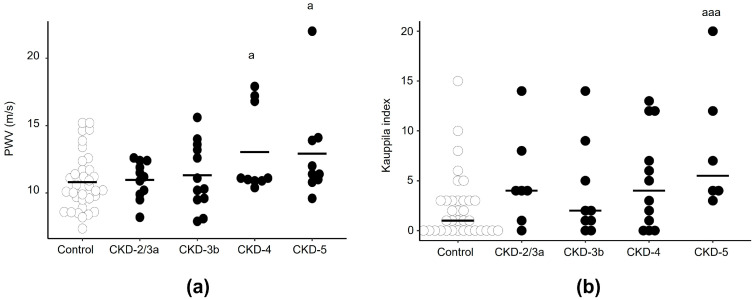
Assessment of vascular damage in the aorta in CKD patients grouped according to eGFR established in the KDIGO guidelines in CKD-2/3a, CKD-3b, CKD-4, and CKD-5 and in sex- and age-matched people from the general population (control group). (**a**) Aortic stiffness measured by pulse wave velocity (PWV) at baseline and (**b**) abdominal aortic quantification by Kauppila index based on a lateral radiograph of the lumbosacral spine after 18-month follow-up. Median and individual values for each group are shown. ^a^ *p* < 0.05 and ^aaa^ *p* < 0.005 versus the control.

At baseline, patients in stage CKD-4 and CKD-5 displayed higher aortic stiffness (Figure 1a). In CKD, aortic stiffness was not correlated to serum levels of sKlotho (r = 0.148; *p* = 0.355) (Supplemental Figure S2a), phosphorus (CKD: r = 0.116; *p* = 0.470), or FGF23 (CKD: r = −0.06; *p* = 0.712). A positive correlation was found between aortic stiffness and serum PTH (r = 0.349; *p* = 0.025), although after adjusting by age, sex, BMI, and eGFR, only age was a predictor of aortic stiffness.

CKD patients displayed no changes in aortic stiffness, assessed by PWV, between baseline and the 18-month follow-up (Supplemental Figure S3).

#### 3.2.2. Kauppila Index

After 18-month follow-up, CKD patients displayed higher KI, reaching the highest values in stage CKD-5 (Figure 1b). Unfortunately, it was not possible to calculate KI at baseline because of technical problems.

No correlation was found between KI and serum levels of Klotho (r = −0.019; *p* = 0.915) (Supplemental Figure S2b), phosphorus (r = 0.029; *p* = 0.870), FGF23 (r = 0.094; *p*= 0.660), or PTH (r = −0.010; *p* = 0.957) in the 18-month follow-up. Furthermore, CKD patients showed a positive correlation between aortic stiffness and KI (r = 0.401; *p* = 0.021), although this correlation was not found after adjusting by age, sex, BMI, and eGFR (β coefficient = 0.086; *p* = 0.288).

#### 3.2.3. Superb Microvascular Image Ultrasound: Atherosclerosis, Carotid Intima–Media Thickness, and Adventitial Neovascularization

##### Atherosclerosis by SMI Ultrasound

At baseline, the proportion of patients with both carotid and calcified carotid plaques was significantly higher in advanced CKD stages (CKD-4 and CKD-5) (Supplemental Table S2). Femoral plaques showed a similar pattern (control: 55.3%; CKD-2/3a: 54.5%; CKD-3b: 83.3%; CKD-4: 90.9%; CKD-5: 77.8%, only statistically significant between CKD-4 and the control, *p* < 0.05).

**Figure 2:**
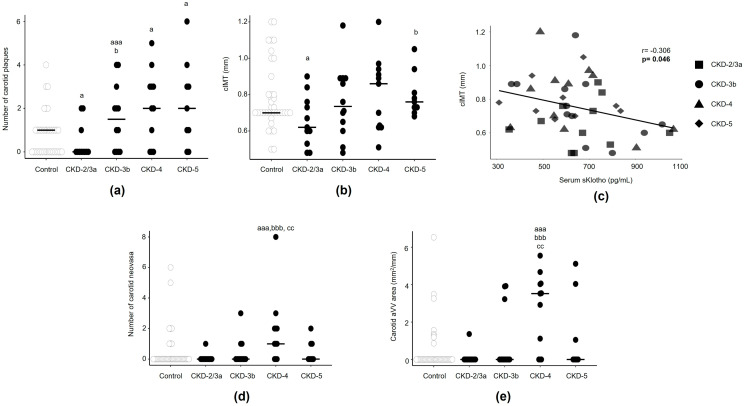
Superb Microvascular Imaging (SMI) ultrasound in carotid artery. (**a**) Number of carotid plaques, (**b**) carotid intima–media thickness (cIMT), (**c**) correlation between serum soluble Klotho (sKlotho) and cIMT, (**d**) number of carotid neovasa, and (**e**) carotid adventitial vasa vasorum (aVV) area in CKD patients grouped according to eGFR established in KDIGO guidelines in CKD-2/3a, CKD-3b, CKD-4, and CKD-5 and in sex- and age-matched people from general population (control group) at baseline. Median and individual values for each group are shown. ^a^ *p* < 0.05, ^aaa^ *p* < 0.005 versus control; ^b^ *p* < 0.05, ^bbb^ *p* < 0.005 versus CKD-2/3a and ^cc^ *p* < 0.01 versus CKD-3b. Each shape represents CKD-2/3a (■), CKD-3b (●), CKD-4 (▲), and CKD-5 (♦).

Furthermore, patients in the CKD-3b, CKD-4, and CKD-5 stages displayed more carotid plaques than the control group (Figure 2a). However, CKD-2/3a displayed fewer carotid plaques than the control group (Figure 2a).

In CKD patients, the number of carotid plaques did not correlate to the serum levels of sKlotho (r = 0.016; *p* = 0.921) (Supplemental Figure S2c), phosphorus (r = 0.157; *p* = 0.314), or FGF23 (r = 0.172; *p* = 0.271), and only a trend was found with serum PTH (r = 0.295; *p* = 0.055).

In the 18-month follow-up, CKD patients displayed more carotid plaques than at baseline, assessed by SMI ultrasound (Supplemental Figure S4).

##### Carotid Intima–Media Thickness by SMI Ultrasound

At baseline, there were no significant increases in any CKD stage compared to the control group, but CKD-5 patients showed higher carotid intima–media thickness compared to those in CKD-2/3a (Figure 2b).

In CKD patients, carotid intima–media thickness was negatively correlated with serum sKlotho levels (r = −0.306; *p* = 0.046). This correlation persisted after adjusting by age, sex, BMI, and eGFR (β coefficient = −0.0004; *p* = 0.007) (Figure 2c). Carotid intima–media thickness was not correlated to serum phosphorus (r = 0.133; *p* = 0.340), FGF23 (r = 0.015; *p* = 0.926), or PTH (r = 0.098; *p* = 0.531) levels.

CKD patients displayed no changes in the carotid intima–media thickness, assessed by SMI ultrasound, between baseline and the 18-month follow-up (Supplemental Figure S5).

##### Adventitial Neovascularization by SMI Ultrasound

In the femoral artery, SMI ultrasound images were not clear enough to detect adventitial neovascularization. Therefore, results are focused only on carotid neovascularization. At baseline, CKD-4 patients showed a higher number of neovasa and area of adventitial neovascularization compared to the controls. In CKD-5, the increase in carotid adventitial neovascularization was not statistically different from the controls (Figure 2d,e).

CKD patients showed no correlation between the area of carotid adventitial neovascularization and serum sKlotho (r = −0.078; *p* = 0.625) (Supplemental Figure S2d), phosphorus (r = 0.215; *p* = 0.171), FGF23 (r = −0.118; *p* = 0.458), or PTH (r = 0.122; *p* = 0.442) levels.

CKD patients displayed no changes in carotid adventitial neovascularization assessed by SMI ultrasound between baseline and the 18-month follow-up (Supplemental Figure S6).

**Figure 3:**
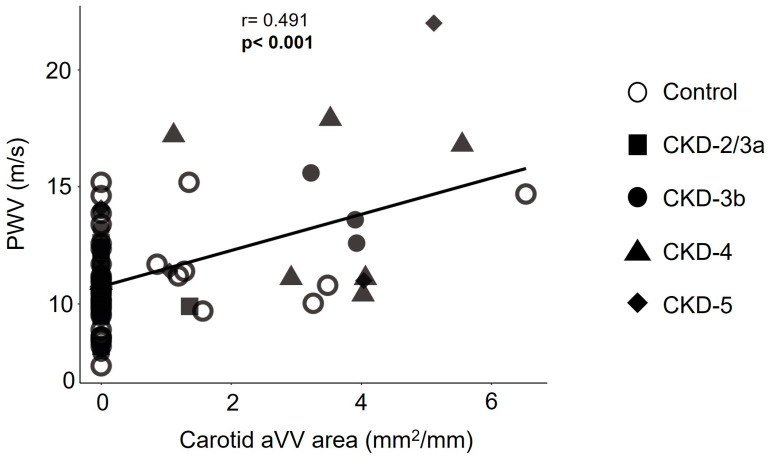
Correlation between carotid adventitial vasa vasorum (aVV) area and pulse wave velocity (PWV) in CKD patients grouped according to eGFR established in KDIGO guidelines in CKD-2/3a, CKD-3b, CKD-4, and CKD-5 and in sex- and age-matched people from general population (control group) at baseline. Each shape represents Control (○), CKD-2/3a (■), CKD-3b (●), CKD-4 (▲), and CKD-5 (♦).

Additionally, considering both the control group and CKD patients, the area of carotid adventitial neovascularization correlated positively with aortic stiffness (r = 0.491; *p* < 0.001) even after adjusting by age, sex, BMI, and eGFR (β coefficient = 0.286; *p* = 0.015) (Figure 3).

In the 18-month follow-up, CKD patients showed no association between adventitial carotid neovascularization and KI.

##### Vascular Damage and Age

At baseline, CKD patients displayed a positive correlation between vascular damage and age: aortic stiffness (r = 0.467; *p* = 0.002), number of carotid plaques (r = 0.486; *p* = 0.001), carotid intima–media thickness (r = 0.482; *p* = 0.001), and area of carotid adventitial neovascularization (r = 0.373; *p* = 0.015). After adjusting by sex, BMI, and eGFR, only the number of carotid plaques (β coefficient = 0.062; *p* = 0.021) and carotid intima–media thickness remained correlated with age (β coefficient = 0.008; *p* = 0.01).

## 4. Discussion

This study aimed to assess vascular damage in patients in stages CKD-2/3a to CKD-5 and its progression after an 18-month follow-up. The applicability of two non-invasive techniques, which have been barely used in CKD patients, was evaluated: (A) carotid–femoral PWV to assess aortic stiffness and (B) SMI ultrasound to assess adventitial neovascularization.

Vascular impairment was detected in advanced CKD stages, normally in CKD-4 and CKD-5. These patients showed higher aortic stiffness, more carotid plaques, higher cIMT, greater adventitial neovascularization, and higher aortic calcification. However, compared with an age- and sex-matched control group, cIMT failed to detect vascular changes. Meanwhile, other non-traditional clinical practice techniques, such as PWV values or adventitial carotid neovascularization, were able to detect vascular impairment in pre-dialysis patients. To our knowledge, this is the first time that an increase in adventitial neovascularization was found in non-dialysis CKD patients. Additionally, CKD patients displayed a negative correlation between serum sKlotho and cIMT. Finally, CKD patients showed no progression of vascular impairment after the 18-month follow-up, except for carotid plaques.

CKD-4 and CKD-5 patients displayed increased PWV values, indicating greater aortic stiffness in advanced CKD stages. It was expected to find higher PWV values at earlier CKD stages, as vascular calcification—the main factor that leads to vascular stiffness—is highly frequent in early CKD [18]. Nevertheless, to our knowledge, just one study has evaluated PWV in the earliest stages of CKD patients finding an early increase in PWV, although compared with a younger, non-age- and sex-matched control group [19].

In addition, after the 18-month follow-up, CKD-5 patients displayed increased PWV and KI, indicating that aortic stiffness might be partially explained by aortic calcification. However, after the adjusted analysis, the correlation was lost, probably influenced by eGFR, although other factors might explain the increased aortic stiffness, such as plaque calcification or arteriosclerosis [5]. In addition, even though carotid–femoral PWV mainly measures aortic stiffness, other arteries could also have influenced the results.

Different studies have suggested aortic PWV as a predictor of CKD progression and mortality [20,21], although they were usually conducted in end-stage renal disease. Although other authors found a slight increase in ankle-brachial and carotid PWV in CKD [22], only carotid–femoral PWV is the gold-standard tool to evaluate aortic stiffness [23].

The present study found no association between PWV values and serum CKD-MBD biomarkers; age was always the main predictor of aortic stiffness. The possible association of PWV with CKD-MBD biomarkers has barely been studied, although PWV might be associated with FGF23 [24] and calcidiol levels [25]. Serum sKlotho did not seem to predict vascular stiffness or calcification, as shown by other studies in pre-dialysis CKD patients [26,27].

Whilst cardiologists sometimes use PWV to complement the values of arterial pressure [11], it is not usually applied in the nephrology field, despite the fact that CKD patients display high cardiovascular risk. Nonetheless, considering the simplicity to perform it and being non-invasive, PWV could help to stratify CKD patients according to their vascular damage and monitor and prevent cardiovascular events before entering dialysis.

With the CKD progression, carotid plaques significantly increased after CKD-3b, as shown by other studies [28,29], supporting the high prevalence of atherosclerosis in CKD [5]. However, CKD did not show statistically significant increased cIMT, suggesting that in our vascularly aged CKD cohort, atherosclerosis is first detected by the number of carotid plaques, making it more difficult to prevent the progression of vascular damage. These results reinforce the importance of having other diagnostic methods to detect vascular damage in pre-dialysis patients. Although cIMT is often considered to be higher in CKD, most studies include a younger control group than the CKD one [30], suggesting that cIMT is more dependent on age than CKD stage.

No association was found among atherosclerosis and serum phosphorus, FGF23, or PTH levels, indicating that, in this CKD cohort, those CKD-MBD parameters were not useful to predict elevated cIMT or plaque prevalence, in accordance with previous studies [31], showing that those parameters might change at a different pace in CKD progression or, maybe, by an effective management of CKD patients. However, recent studies found that CKD-MBD biomarkers were related to atherosclerosis [32] probably by acting directly on the vasculature [33] or through hypertrophic and proinflammatory mechanisms [34].

Serum sKlotho was the only CKD-MBD biomarker that was associated with cIMT in CKD patients, which is in agreement with previous studies [35,36]. sKlotho was shown to exert protective actions on the vasculature, preventing vascular calcification, inhibiting endothelial inflammation, reducing apoptosis, or inducing autophagy [37,38,39,40]. This could partially explain why CKD patients with higher serum sKlotho showed thinner cIMT, but other factors might have been relevant, such as aging [41] or inflammation [42]. Very recently, serum sKlotho levels under 760 pg/mL were suggested to predict cardiovascular mortality in CKD [43], supporting the above-mentioned causality. This evidence strengthens the potential application of serum sKlotho as a biomarker of vascular damage in CKD.

To evaluate adventitial neovascularization, an SMI ultrasound was performed both in carotid and femoral arteries. However, it was not possible to precisely determine femoral neovascularization probably due to the complexity to perform the analysis because of the curve-shaped structure of this artery [44].

CKD-4 patients displayed increased carotid adventitial neovascularization, indicating greater vascular impairment and risk of developing more carotid plaques. Normally, big arteries display a network of vasa vasorum that nourish the vascular wall. However, under ischemia or systemic inflammation, so frequent in CKD, new vasa vasorum—neovascularization—is generated, leading to damage and inflammation, as they promote the infiltration of inflammatory cells [45].

Adventitial neovasa were suggested to appear even before atherosclerosis in the general population, or before coronary artery disease and diabetes [10,46,47], which is associated with CKD [48]. Although already used to detect plaque neovascularization [14], SMI ultrasound remains completely experimental to assess adventitial neovascularization.

Our group previously detected increased carotid adventitial neovascularization in patients undergoing peritoneal dialysis [15], but, to our knowledge, this is the first time to demonstrate increased neovascularization in pre-dialysis CKD patients, suggesting that the non-invasive SMI might be useful to stratify patients according to their vascular impairment before entering dialysis, being more precise than cIMT, which is normally used in clinical practice. As far as we are concerned, the possible association between microvascularization detected by SMI and CKD-MBD biomarkers has not been previously investigated. Nevertheless, SMI was indeed used in the nephrology field, but with a completely different approach, as it has been used to to evaluate renal disfunction and indeterminate renal masses [49,50].

Unexpectedly, CKD-5 patients did not display statistically significant increased carotid adventitial neovascularization probably because of the smaller sample size, the fewer proportion of pharmacologically treated patients, or the higher proportion of women in this group, suggesting a protective effect of estrogens against vascular damage [51], although there are no data on sex-specific differences. In fact, women in the control group displayed lower systolic blood pressure, thinner cIMT, and less area of carotid adventitial neovascularization. However, in CKD, the previously mentioned parameters showed no differences between men and women, indicating that CKD-induced damage might overcome the protection induced by estrogens, although other authors have observed less cardiovascular risk even in women with CKD [52].

Interestingly, PWV values and carotid adventitial neovascularization were positively correlated, suggesting reliability in detecting vascular damage. Both tools identified two types of vascular damage simultaneously, strengthening their diagnostic value and setting a potential connection between two pathological mechanisms: adventitial neovascularization might promote plaque formation, leading to higher aortic stiffness, although this biological link remains to be elucidated.

PWV and SMI ultrasound detected vascular damage at different stages during CKD progression: while the number of carotid plaques increased in CKD-3b, the increase in aortic stiffness, cIMT, and carotid adventitial neovascularization was detected in the CKD-4 or CKD-5 stages. Therefore, carotid plaques might be the earliest vascular damage to be detected in CKD, with the limitation that it indicates that atherosclerosis is already established.

In the 18-month follow-up, only the number of carotid plaques showed an increase in CKD patients, indicating rapid progression of CKD-related atherosclerosis [53]. No increase was found in aortic stiffness after 18-months, so vascular calcification might be a slower process than atherosclerosis, although some studies found that CKD patients, after a one-year follow-up, showed an increment of 1 m/s in PWV [24,54]. So far, no other study has evaluated the progression of carotid adventitial neovascularization in CKD, although it might change rapidly under statin treatment [55]. A reason for the lack of vascular damage progression between baseline and the 18-month follow-up could be the loss of several patients that entered dialysis and were excluded from the follow-up, as those patients might have been the ones with the fastest progression of vascular damage.

Aging is a major predictor of vascular damage and in CKD, vascular aging is highly accelerated [56]. Our CKD cohort showed a clear and expected correlation between vascular parameters and age. However, apart from serum sKlotho and cIMT, no associations were found between vascular damage and serum CKD-MBD biomarkers, which were usually overcome by age. Aging could also partially explain the late detection of vascular damage in CKD, as the control group showed considerable age-related vascular damage.

Most studies evaluating cardiovascular damage in CKD were conducted in considerably aged patients and, although some of them did find vascular damage in early stages of the disease, they were mainly focused on atherosclerosis, cardiovascular events, or mortality [53,57], and not on the early and non-invasive detection of vascular damage.

Curiously, when it came to cIMT and carotid adventitial neovascularization, CKD-2/3a patients displayed less vascular damage than the control group probably because of the effect of age and clinical management in vascular damage: CKD-2/3a patients were slightly younger and probably under clinical supervision, leading to improved vascular health and reduced prevalence of carotid plaques.

Could PWV and SMI ultrasound improve current clinical management in CKD? Currently, the most used methods to evaluate cardiovascular risk are cIMT and plaque detection by ultrasound and coronary calcium assessed by computed tomography [58]. Carotid evaluation by ultrasound is considerably simple to perform, reproducible, cheap, and with great predictive value. However, in our population of study, PWV and SMI were the ones to detect vascular damage in CKD. Furthermore, coronary calcium assessment displays great predictive value, but it is more complex to perform, as well as being expensive and invasive. In contrast, PWV and SMI are both considerably simple to perform and cheaper. Finally, X-rays of the abdominal aorta are also use to assess vascular damage—calcification—but it is an invasive test, as it requires radiation [3].

In addition, cardiovascular risk stratification is based on validated methods like SCORE2, PCE, Framingham Risk Score, ASCVD Risk Calculator, and QRISK3. These scores estimate 10-year cardiovascular event risks based on multiple factors. Each tool has specific focuses and limitations, with recent guidelines advocating additional risk modulators. Limitations include the lack of external validation and miscalculation in diverse populations [59].

### Limitations and Strengths

This study presents some limitations. The high mean age of the participants could have influenced the late detection of vascular impairment in the progression of CKD. Additionally, results should be confirmed in a larger cohort, focusing on comparing different CKD stages. The lack of KI values at baseline does not allow for the assessment of the possible progression of aortic calcification in CKD. Furthermore, the detection of adventitial neovascularization was not confirmed using a standardized ultrasound technique, such as contrast enhanced ultrasound. In addition, both PWV and SMI ultrasound present a few limitations. As for PWV, the assessed distance between the carotid and femoral arteries might vary depending on the operator and branched arteries along the aorta might influence the results [23]. Regarding SMI ultrasound, as it is a relatively new technology, there are no standardized methods to quantify neovascularization and there is limited background evidence [60].

Nevertheless, this work did have some strong points. The applicability of novel non-invasive tools—PWV and SMI ultrasound—was evaluated to measure vascular damage in CKD and it was the first time increased carotid adventitial neovascularization in pre-dialysis patients by SMI ultrasound was assessed. Both PWV and adventitial neovascularization showed the advantage of detecting vascular damage in pre-dialysis patients before other markers often used in clinical practice, such as cIMT. In addition, PWV and SMI ultrasound technologies are cheaper than other cardiovascular diagnostic tools—SMI does not require the use of a contrast agent—both tests are considerably fast to perform—around 20 min each—and the equipment is easily installed in a regular medical office, paving the way for their routinary use [9,23]. Additionally, with brief and practical training, most healthcare professionals can acquire the necessary skills to use these tools effectively. More specifically, performing PWV requires basic knowledge to identify pulse in the carotid and femoral arteries to place the sensors. Ideally, two people are required to make the assessment, as it is complex to handle both sensors at the same time. Finally, to ensure reproducibility, the test should always be performed by the same two operators. As for SMI, it is an ultrasound tool and, therefore, any trained radiologist should be able to perform this test correctly.

## 5. Conclusions

In advanced CKD stages, significant vascular impairment was detected using non-traditional techniques. Traditional cIMT failed to identify changes, but PWV and adventitial neovascularization revealed vascular alterations. This study uniquely identified adventitial neovascularization in non-dialysis CKD patients, with a negative correlation between sKlotho and cIMT. No major vascular progression occurred over 18 months, except for carotid plaque development. In conclusion, performing non-invasive PWV and SMI ultrasound might be more useful than other diagnostic methods, such as cIMT, to evaluate vascular damage in CKD patients before entering dialysis, possibly helping to prevent cardiovascular events. These tools did not seem to detect the progression of aortic stiffness and carotid adventitial neovascularization in a short-term follow-up. Future clinical studies might clarify the use of these techniques in the clinical practice, their possible association with serum CKD-MBD biomarkers, and the pathophysiological origin of adventitial neovascularization in CKD.

## Figures and Tables

**Table 1:** Anthropometric, clinical, and biochemical parameters and pharmacological treatments in CKD patients and control group.

	Control (n = 38)	CKD-2/3a (n = 11)	CKD-3b (n = 12)	CKD-4 (n = 11)	CKD-5 (n = 9)
Anthropometric and clinical features					
Age (years)	67 ± 5	62 ± 10	67 ± 7	69 ± 9	69 ± 6
Sex (%)	H: 47 M: 53	H: 55 M: 45	H: 75 M: 25	H: 73 M: 27	H: 22 M: 78
BMI (kg/m^2^)	27 ± 5	31 ± 5	27 ± 3	31 ± 7	27 ± 4
Systolic blood pressure (mm Hg)	132 [121–143]	140 [136–142]	135 [128–141]	163 [142–176] ^aaa,c^	138 [126–172]
Dyastolic blood pressure (mm Hg)	75 ± 10	84 ± 13	76 ± 13	82 ± 15	74 ± 12
Biochemical parameters					
eGFR (mL/min/1.73 m^2^)	82 [75–88]	49 [47–53] ^aaa^	39 [34–40] ^aaa^	23 [21–25] ^aaa,b^	12 [12,13] ^aaa,bbb,c^
Creatinine (mg/dL)	0.8 [0.7–1.0]	1.2 [1.2–1.5] ^aaa^	1.8 [1.6–1.8] ^aaa^	2.7 [2.3–3.0]^aaa,b^	3.7 [3.4–4.5] ^aaa,bbb,c^
Total protein (g/L)	70.7 ± 3.5	71.2 ± 3.1	69.5 ± 2.9	69.1 ± 3.6	68.5 ± 6.0
Calcium (mg/dL)	9.5 ± 0.3	9.5 ± 0.3	9.6 ± 0.4	9.7 ± 0.6	9.4 ± 0.5
Phosphorus (mg/dL)	3.6 [3.5–4.0]	3.2 [3.5–3.5] ^a^	3.3 [2.7–3.5] ^aaa^	3.4 [3.1–3.9]	4.1 [3.8–4.9] ^a,bbb,ccc,ddd^
PTH (pg/mL)	52 [42–61]	56 [43–75]	87 [72–118] ^aaa^	114 [72–199] ^aaa,bb^	176 [155–212] ^aaa,bbb,c^
Calcitriol (pg/mL)	46 ± 12	39 ± 12	29 ± 10 ^aaa^	35 ± 14	24 ± 12 ^aaa, b^
Calcidiol (ng/mL)	29 ± 13	31 ± 15	25 ± 10	27± 7	23 ± 12
FGF23 (pg/mL)	55 [46–65]	57 [52–63]	135 [81–166] ^aaa,b^	144 [101–241] ^aaa,bb^	176 [141–1689] ^aaa,bbb^
sKlotho (pg/mL)	809 [680–1042]	671 [604–746] ^a^	632 [600–713] ^aa^	591 [517–707] ^aaa^	586 [469–674] ^aaa^
Urinary creatinine (mg/dL)	106 [75–151]	45 [35–101] ^a^	96 [58–114]	83 [57–96]	47 [47–71] ^aaa^
Proteinuria/urinary creatinine (mg/dL)	0.06 [0.05–0.08]	0.09 [0.05–0.15]	0.40 [0.12–0.84] ^aaa,b^	0.41 [0.15–1.20] ^aaa^	0.81 [0.43–2.12] ^aaa,bbb^
Treatments (% of individuals)					
Statins	13	14	26	26	17
Antihypertensives	18	12	21	26	17
Vitamin D Supplementation	21	7	9	14	7
Paricalcitol	0	0	0	12	9

CKD, Chronic Kidney Disease; BMI, body mass index; eGFR, estimated glomerular filtration rate; PTH, parathyroid hormone; FGF23, fibroblast growth factor 23; sKlotho, soluble Klotho. Vitamin D supplementation (cholecalciferol or calcidiol). ^a^ *p* < 0.05, ^aa^ *p* < 0.01, and ^aaa^ *p* < 0.005 vs. Control group; ^b^ *p* < 0.05, ^bb^ *p* < 0.01, and ^bbb^ *p* < 0.005 vs. CKD-2/3a, ^c^ *p* < 0.05 and ^ccc^ *p* < 0.005 vs. CKD-3b and ^ddd^ *p* < 0.005 vs. CKD-4. Values are expressed as median [interquartile range] or mean ± standard deviation according to data distribution. Kruskal–Wallis or ANOVA and Dunn or Tukey tests as post hoc analysis were used as statistical methods.

## Data Availability

The data underlying this article will be shared upon reasonable request to the corresponding authors.

## Supplementary Materials

The following supporting information can be downloaded at: https://www.mdpi.com/article/10.3390/biomedicines13030621/s1. Supplemental Table S1: Anthropometric, clinical, and biochemical parameters and pharmacological treatments in CKD patients and the control group at the 18-month follow-up. Supplemental Table S2: Percentage of individuals with carotid plaques. Supplemental Figure S1: SMI ultrasound example images. (A) Subject with no adventitial neovascularization and (B) subject with adventitial neovascularization (see arrow). Each inset indicates relative scale in mm. Supplemental Figure S2: Correlations among serum soluble Klotho (sKlotho) and (A) pulse wave velocity (PWV), (B) Kauppila index, (C) number of carotid plaques, and (D) carotid adventitial vasa vasorum (aVV) area in CKD patients at baseline or 18-month of follow-up. Supplemental Figure S3: Progression of aortic stiffness measured by pulse wave velocity (PWV) in CKD patients after 18-month of follow-up. Supplemental Figure S4: Progression of number of carotid plaques measured by Superb Microvascular Imaging (SMI) in CKD patients after 18-month of follow-up. Supplemental Figure S5: Progression of carotid intima–media thickness (cIMT) by Superb Microvascular Imaging (SMI) in CKD patients after 18-month of follow-up. Supplemental Figure S6: Progression of adventitial neovascularization by Superb Microvascular Imaging (SMI). (A) Number of carotid neovasa and (B) carotid adventitial vasa vasorum (aVV) area in CKD patients after 18-month of follow-up.

## Abbreviations

The following abbreviations are used in this manuscript:

CKD	Chronic Kidney Disease
CKD-MBD	Chronic Kidney Disease–Mineral Bone Disorder
PTH	Parathyroid hormone
FGF23	Fibroblast Growth Factor 23
sKlotho	Soluble Klotho
KI	Kauppila index
cIMT	Carotid intima–media thickness
CEUS	Contrast enhanced ultrasound
PWV	Pulse wave velocity
SMI	Superb Microvascular Imaging
BMI	Body mass index
eGFR	Estimated glomerular filtration rate
