# Supplementary material for: Non-Invasive Assessment of Vascular Damage Through Pulse Wave Velocity and Superb Microvascular Imaging in Pre-Dialysis Patients
Source: Biomedicines. 2025 Mar 4;13(3):621. doi: 10.3390/biomedicines13030621 (PMC11940463; doi:10.3390/biomedicines13030621)
Supplement: Supplementary file 1 [file biomedicines-13-00621-s001.zip › biomedicines-3443044-supplementary.pdf]

Supplementary Material

- **Table S1:** Anthropometric, clinical, biochemical parameters and pharmacological treatments in CKD patients and the control group at the 18-month follow-up.

- **Table S2:** Percentage of individuals with carotid plaques.

- **Figure S1**: SMI ultrasound example images. A) Subject with no adventitial neovascularization and B) Subject with abundant adventitial neovascularization. Each inset indicates the relative scale in mm.

- **Figure S2:** Correlations between serum soluble Klotho (sKlotho) and A) pulse wave velocity (PWV), B) Kauppila Index, C) number of carotid plaques and D) carotid adventitial vasa vasorum (aVV) area in CKD patients grouped according to eGFR stablished in the KDIGO guidelines in CKD-2/3a, CKD-3b, CKD-4 and CKD-5 at baseline or 18-month of follow-up. Each shape represents CKD-2/3a (■), CKD -3b (●), CKD -4 (▲) and CKD -5 (♦).

- **Figure S3:** Progression of aortic stiffness measured by pulse wave velocity (PWV) in CKD patients grouped according to eGFR stablished in the KDIGO guidelines in CKD-2/3a, CKD-3b, CKD-4 and CKD-5 after the 18-month of follow-up. Median and individual values for each group are shown. The progression of each patient is shown by a grey line. CKD basal: CKD patients at baseline, CKD 18: CKD patients after 18-month follow-up.

- **Figure S4:** Progression of number of the carotid plaques measured by superb microvascular imaging (SMI) in CKD patients grouped according to eGFR stablished in the KDIGO guidelines in CKD-2/3a, CKD-3b, CKD-4 and CKD-5 after the 18-month of follow-up. Median and individual values for each group are shown. The progression of each patient is shown by a grey line. CKD basal: CKD patients at baseline, CKD 18: CKD patients after 18-month follow-up.

- **Figure S5:** Progression of carotid intima-media thickness (cIMT) by superb microvascular imaging (SMI) in CKD patients grouped according to eGFR stablished in the KDIGO guidelines in CKD-2/3a, CKD-3b, CKD-4 and CKD-5 after the 18-month of follow-up. Median and individual values for each group are shown. The progression of each patient is shown by a grey line. CKD basal: CKD patients at baseline, CKD 18: CKD patients after 18-month follow-up.

- **Figure S6:** Progression of adventitial neovascularization by superb microvascular imaging (SMI). A) Number of carotid neovasa and B) carotid adventitial vasa vasorum (aVV) area in CKD patients grouped according to eGFR stablished in the KDIGO guidelines in CKD-2/3a, CKD-3b, CKD-4 and CKD-5 after the 18-month of follow-up. Median and individual values for each group are shown. The progression of each patient is shown by a grey line. CKD basal: CKD patients at baseline, CKD 18: CKD patients after 18-month follow-up.

Table S1. Anthropometric, clinical, biochemical parameters and pharmacological treatments in CKD patients and the control group at the 18-month follow-up.

	Control (n=38)	CKD-2/3a (n=11)	CKD-3b (n=12)	CKD-4 (n=11)	CKD-5 (n=9)
**Anthropometric and clinical features**					
**Age (years)**	68±5	68±6	66±10	69±9	72±8
**Sex (%)**	H: 47 M: 53	H: 57 M: 43	H: 44 M: 56	H: 77 M: 23	H: 33 M: 67
**BMI (kg/m^2)**	27±5	30±3	29±4	27±5	27±3
**Systolic blood pressure (mm Hg)**	128 [120-134]	132 [119-134.5]	134 [133-142]	139 [115-154]	137 [131-165]
**Dyastolic blood pressure (mm Hg)**	73±10	73±10	70±15	74±18	79±6
**Biochemical parameters**					
**eGFR (mL/min/1,73 m^2)**	83 [77-87]	**54 [46-57]**[a]	**39 [36-40]**[aaa]	**23 [20-26]**[aaa, b]	**10 [9-11]**[aaa, b]
**Creatinine (mg/dL)**	0.8 [0.7-1]	**1.2 [1.1-1.4]**[a]	**1.5 [1.4-1.8]**[aaa]	**2.6 [2.5-2.9]**[aaa, b]	**4.8 [4.1-5.3]**[aaa, b]
**Total protein (g/L)**	69.0±3.6	70.8±3.7	62.1±25.2	70.2±4.2	69.4±3.3
**Calcium (mg/dL)**	9.5±0.3	9.5±0.3	9.8±0.4	9.7±0.5	9.5±0.5
**Phosphorus (mg/dL)**	3.7 [3.4-3.8]	3.5 [3.4 -3.6]	3.6 [3.0- 3.6]	3.4 [3.4 -3.6]	**4.5 [4.1 - 4.7]**[aaa,bbb,ccc, ddd]
**PTH (pg/mL)**	51 [41-62]	58 [48-75]	**70 [66-91]**[a]	**108 [91-192]**[aaa,bb]	**180 [158-191]**[aaa, bbb, c]
**Calcitriol (pg/mL)**	43±12	41±19	**28±6** [a]	**28±16** [a]	**31± 22**
**Calcidiol (ng/mL)**	29±12	43±19	26±9	29±9	29± 19
**FGF23 (pg/mL)**	59 [47 - 81]	77 [70-80]	**99 [57-161]**[a]	**242 [123- 286]**[aaa]	**501 [384-607]**[aaa,bbb,ccc]
**sKlotho (pg/mL)**	844 [740-1030]	785 [746-1001]	699 [649- 931]	**752 [654-837]**[a]	**630 [519-720]**[aaa,b]
**Urinary creatinine (mg/dL)**	129 [88-153]	81 [78-114]	**60 [46-70]**[aaa]	**80 [52-100]**[aaa]	**64 [47 -112]**[a]
**Proteinuria/urinary creatinine (mg/dL)**	0.06 [0.05-0.08]	0.09 [0.06-0.17]	**0.18 [0.09-0.26]**[a, b]	**0.59 [0.13- 1.05]**[aaa]	**0.97 [0.48-1.55]**[aaa, b,c]
**Treatments (% of individuals)**					
**Statins**	21	17	20	34	17
**Antihypertensives**	19	11	17	31	17
**Native Vitamin D**	28	14	11	29	11
**Paricalcitol**	3	0	3	11	11

CKD, Chronic Kidney Disease; BMI, Body Mass Index; eGFR, estimated glomerular filtration rate; PTH, parathyroid hormone; FGF23, fibroblast growth factor 23; sKlotho, soluble Klotho. ap<0.05, aap<0.01 and aaap<0.005 vs. Control group; bp<0.05, bbp<0.01 and bbbp<0.005 vs. CKD-2/3a, cp<0.05 and cccp<0.005 vs. CKD-3b and dddp<0.005 vs. CKD-4. Values are expressed as Median [interquartile range] or mean±standard deviation according to data distribution. Kruskal-Wallis or ANOVA and Dunn or Tukey tests as post hoc analysis were used as statistical methods.

Table S2. Percentage of individuals with carotid plaques.

	Individuals with carotid plaques (%)	Individuals with calcified carotid plaques (%)
**Control**	47.4	34.2
**CKD-2/3a**	27.3	9.1
**CKD-3b**	66.7	41.7
**CKD-4**	**72.7**[b]	**54.5** [b]
**CKD-5**	**77.8** [b]	**55.6** [b]

CKD, Chronic Kidney Disease. bp<0.05 vs. CKD-2/3a.

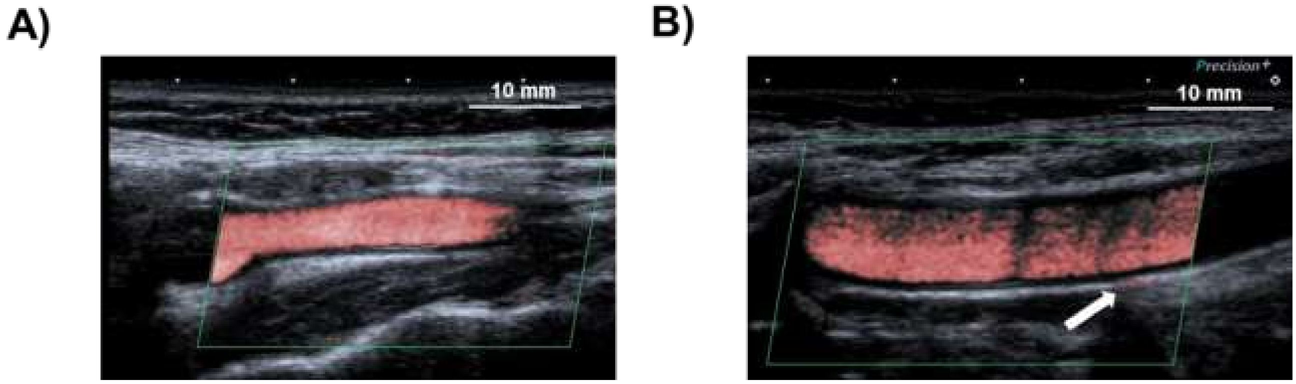

Figure S1: SMI ultrasound example images. A) Subject with no adventitial neovascularization and B) Subject with adventitial neovascularization (see arrow). Each inset indicates the relative scale in mm.

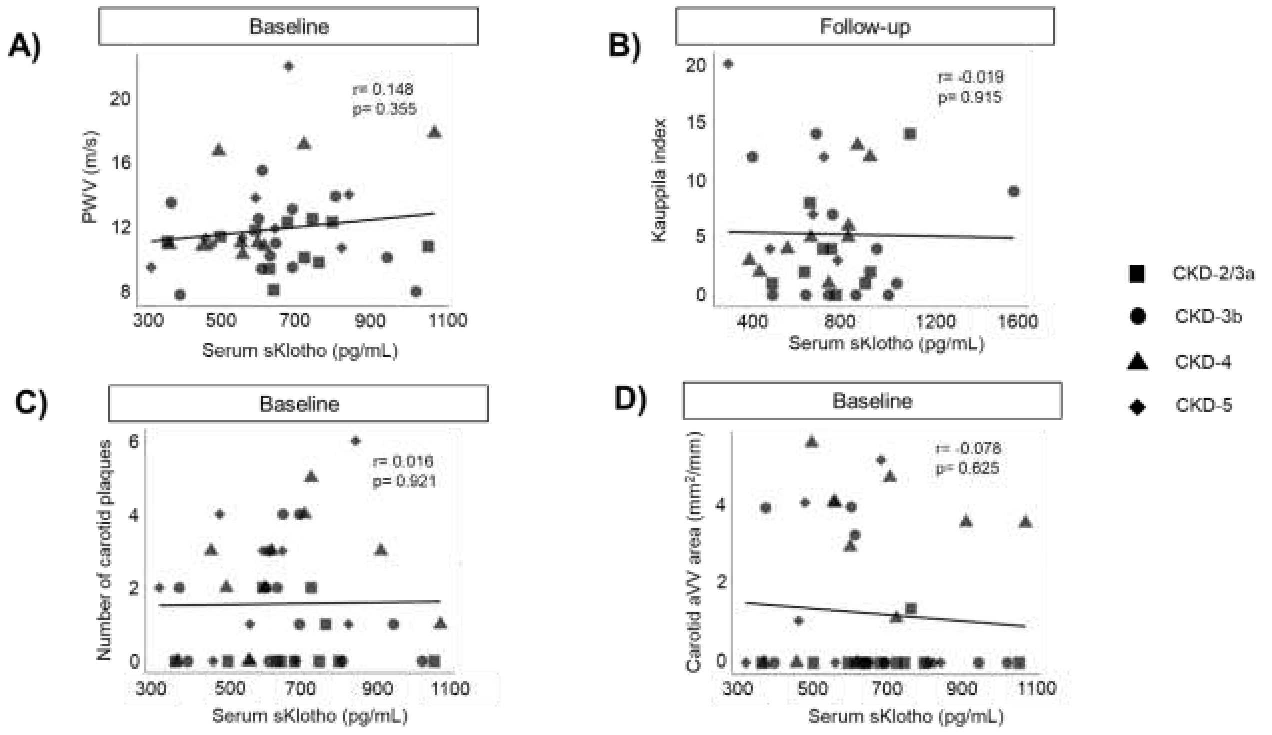

Figure S2: Correlations between serum soluble Klotho (sKlotho) and A) pulse wave velocity (PWV), B) Kauppila Index, C) number of carotid plaques and D) carotid adventitial vasa vasorum (aVV) area in CKD patients grouped according to eGFR stablished in the KDIGO guidelines in CKD-2/3a, CKD-3b, CKD-4 and CKD-5 at baseline or 18-month of follow-up. Each shape represents CKD-2/3a (■), CKD -3b (●), CKD -4 (▲) and CKD -5 (♦).

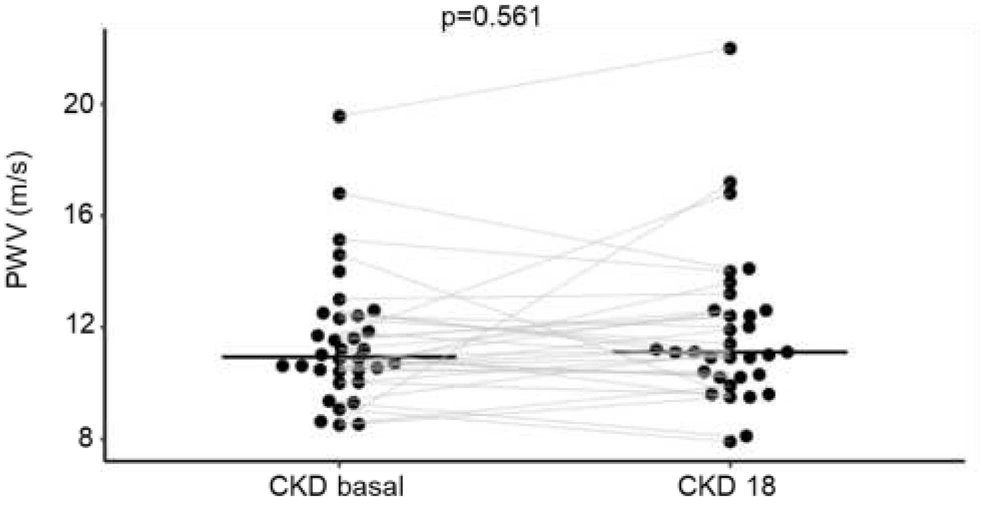

Figure S3: Progression of aortic stiffness measured by pulse wave velocity (PWV) in CKD patients grouped according to eGFR stablished in the KDIGO guidelines in CKD-2/3a, CKD-3b, CKD-4 and CKD-5 after the 18-month of follow-up. Median and individual values for each group are shown. The progression of each patient is shown by a grey line. CKD basal: CKD patients at baseline, CKD 18: CKD patients after 18-month follow-up.

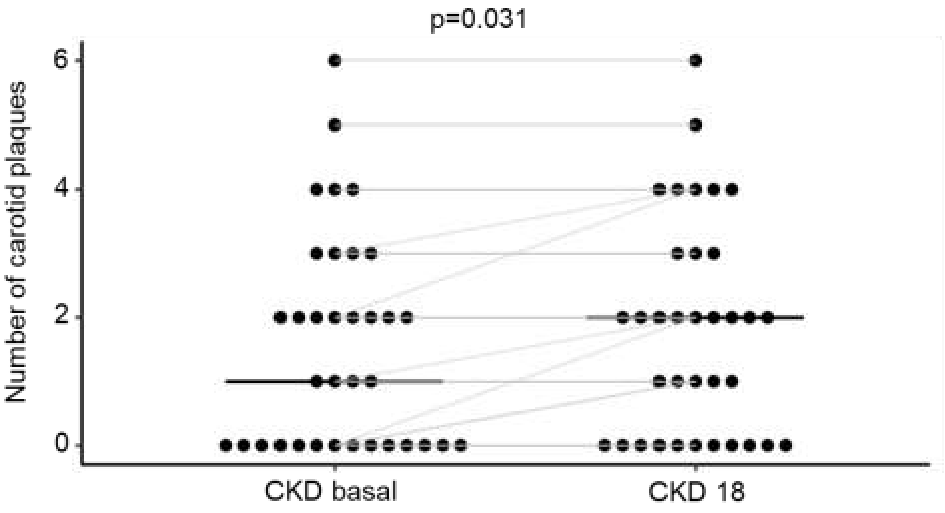

Figure S4: Progression of number of the carotid plaques measured by superb microvascular imaging (SMI) in CKD patients grouped according to eGFR stablished in the KDIGO guidelines in CKD-2/3a, CKD-3b, CKD-4 and CKD-5 after the 18-month of follow-up. Median and individual values for each group are shown. The progression of each patient is shown by a grey line. CKD basal: CKD patients at baseline, CKD 18: CKD patients after 18-month follow-up.

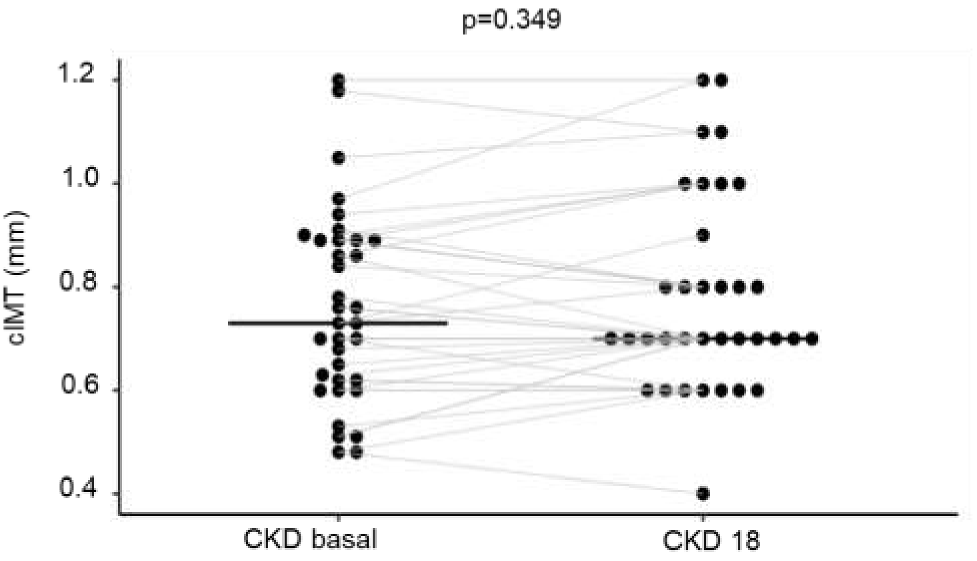

Figure S5: Progression of carotid intima-media thickness (cIMT) by superb microvascular imaging (SMI) in CKD patients grouped according to eGFR stablished in the KDIGO guidelines in CKD-2/3a, CKD-3b, CKD-4 and CKD-5 after the 18-month of follow-up. Median and individual values for each group are shown. The progression of each patient is shown by a grey line. CKD basal: CKD patients at baseline, CKD 18: CKD patients after 18-month follow-up.

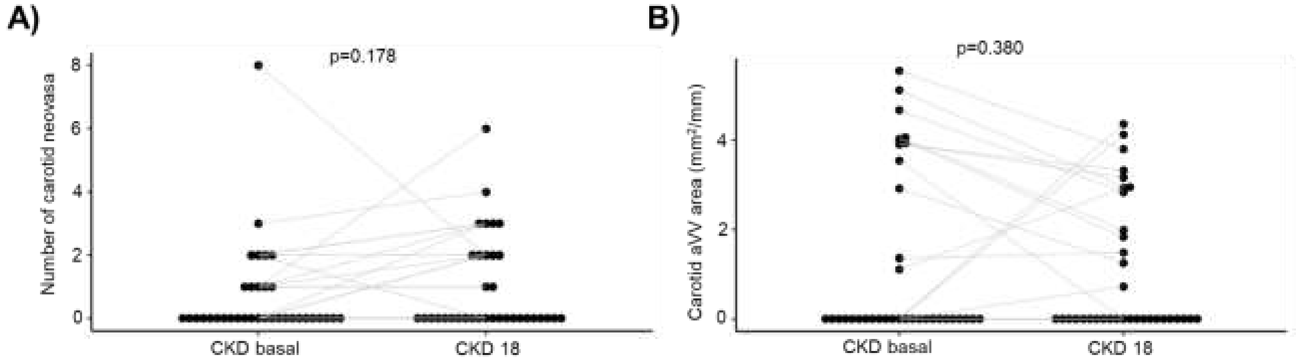

Figure S6: Progression of adventitial neovascularization by superb microvascular imaging (SMI). A) Number of carotid neovasa and B) carotid adventitial vasa vasorum (aVV) area in CKD patients grouped according to eGFR stablished in the KDIGO guidelines in CKD-2/3a, CKD-3b, CKD-4 and CKD-5 after the 18-month of follow-up. Median and individual values for each group are shown. The progression of each patient is shown by a grey line. CKD basal: CKD patients at baseline, CKD 18: CKD patients after 18-month follow-up.
